# Performance of 3D printed porous polyetheretherketone composite scaffolds combined with nano-hydroxyapatite/carbon fiber in bone tissue engineering: a biological evaluation

**DOI:** 10.3389/fbioe.2024.1343294

**Published:** 2024-01-25

**Authors:** Lian Mi, Feng Li, Dian Xu, Jian Liu, Jian Li, Lingmei Zhong, Yanshan Liu, Na Bai

**Affiliations:** ^1^ Department of Oral Prosthodontics, The Affiliated Hospital of Qingdao University, Qingdao, China; ^2^ School of Stomatology, Qingdao University, Qingdao, China; ^3^ State Key Laboratory of Oral and Maxillofacial Reconstruction and Regeneration, National Clinical Research Center for Oral Diseases, Shaanxi Clinical Research Center for Oral Diseases, Department of Oral and Maxillofacial Surgery, School of Stomatology, The Fourth Military Medical University, Xi’an, China; ^4^ Department of Pulmonary and Critical Care Medicine, The Affiliated Hospital of Qingdao University, Qingdao, China; ^5^ Department of Oral and Maxillofacial Surgery, The Affiliated Hospital of Qingdao University, Qingdao, China; ^6^ Dental Digital Medicine and 3D Printing Engineering Laboratory of Qingdao, Qingdao, China

**Keywords:** polyetheretherketone (PEEK), composite materials, fused deposition molding (FDM), bone tissue engineering, porous scaffolds

## Abstract

Polyetheretherketone (PEEK) has been one of the most promising materials in bone tissue engineering in recent years, with characteristics such as biosafety, corrosion resistance, and wear resistance. However, the weak bioactivity of PEEK leads to its poor integration with bone tissues, restricting its application in biomedical fields. This research effectively fabricated composite porous scaffolds using a combination of PEEK, nano-hydroxyapatite (nHA), and carbon fiber (CF) by the process of fused deposition molding (FDM). The experimental study aimed to assess the impact of varying concentrations of nHA and CF on the biological performance of scaffolds. The incorporation of 10% CF has been shown to enhance the overall mechanical characteristics of composite PEEK scaffolds, including increased tensile strength and improved mechanical strength. Additionally, the addition of 20% nHA resulted in a significant increase in the surface roughness of the scaffolds. The high hydrophilicity of the PEEK composite scaffolds facilitated the *in vitro* inoculation of MC3T3-E1 cells. The findings of the study demonstrated that the inclusion of 20% nHA and 10% CF in the scaffolds resulted in improved cell attachment and proliferation compared to other scaffolds. This suggests that the incorporation of 20% nHA and 10% CF positively influenced the properties of the scaffolds, potentially facilitating bone regeneration. *In vitro* biocompatibility experiments showed that PEEK composite scaffolds have good biosafety. The investigation on osteoblast differentiation revealed that the intensity of calcium nodule staining intensified, along with an increase in the expression of osteoblast transcription factors and alkaline phosphatase activities. These findings suggest that scaffolds containing 20% nHA and 10% CF have favorable properties for bone induction. Hence, the integration of porous PEEK composite scaffolds with nHA and CF presents a promising avenue for the restoration of bone defects using materials in the field of bone tissue engineering.

## 1 Introduction

Tumors, trauma, and infection are common causes of irregular bone defects or comminuted fractures in oral and maxillofacial surgery. Despite the ability of bone tissue to heal itself, it remains a significant challenge for clinicians to repair complex bone defects. Clinically, autogenous bone is the most commonly used, but it has some drawbacks, including limited donor sources, higher patient pain, and a longer healing time. Biomaterials such as biometals, bioceramics, and biopolymers are commonly used in bone implants in clinical practice, but their application is limited (Wang C et al., 2020; Tan et al., 2021; Xue et al., 2022). The modulus of elasticity of biometals and alloys is much greater than the modulus of elasticity of bone tissue, which produces a stress shielding effect on bone tissue (Sukotjo et al., 2020; Sun et al., 2021; Wang et al., 2021; Zhuang et al., 2021). Bioceramics have strong bioactivity augmentation that can be used to boost polymer osteogenic activity. Due to their biological fragility and unstable biodegradability, they are mostly used as bone filler materials (Qian et al., 2021; Qian et al., 2024). Polymers, including natural and synthetic polymers, offer a wide range of sources, including biocompatibility, osteoconductivity, low immunogenicity, and biodegradability, and some of these materials have been approved for clinical application. Although these materials have flaws, such as a quick degradation rate and irritation of degradation products that cause inflammatory reactions, it is still expected that they can be enhanced for use in bionic structures. Bone tissue engineering is a technique that utilizes the principles and methods of biology, engineering, and materials science to repair and regenerate damaged bone tissue. It facilitates the proliferation and differentiation of bone cells by producing biocompatible materials and cellular scaffolds (Lewns et al., 2023) and enables customized treatment according to the condition of the patient (Jia et al., 2023). The ideal material for bone tissue engineering needs to fulfill various requirements such as printability, biocompatibility, mechanical properties, and bioactivity. However, bone tissue engineering is still in the research stage, and the ideal material has not yet been found. Therefore, a novel bone tissue engineering material that performs excellently is urgently needed.

Polyetheretherketone (PEEK) is becoming widely studied in the medical field, according to recent research. PEEK is a thermoplastic aromatic polymer with ether and ketone interactions, which provide it with excellent physical and chemical properties. PEEK is excellent in terms of biocompatibility, high temperature resistance, aging resistance, and ease of processing. Furthermore, PEEK has a modulus of elasticity that is closer to that of human bone, reducing bone resorption. The most notable feature of PEEK compared to other bone graft materials is its transmission linearity. It is invisible during radiographic examinations, which allows for better visualization of bone tissue. Currently, PEEK is used in the biomedical field as an implant for reconstruction of larger defects such as cervical and lumbar fusions, cranial defects, and orbital defects (He et al., 2019; Ling et al., 2020; Wei et al., 2023). However, PEEK’s weak bioactivity results in poor bonding with bone tissue (Chen et al., 2022), restricting its widespread use in biomedical applications. Previous studies have shown that the porous structure of 3D printing makes it easier for cells and blood vessels to enter the pores (Juan et al., 2021; Lei et al., 2022). Selective laser sintering (SLS) and fused deposition modeling (FDM) are two common processes for 3D printing PEEK. However, SLS technology is quite expensive and wastes a lot of raw material during the printing process, necessitating residue powder cleanup (Qian et al., 2023b). FDM is gradually replacing this technique. By melting materials into a fused state and layering them, it enables the production of three-dimensional objects. FDM has the advantages of quick production, low cost, and flexible design. It was discovered by Torstrick et al. (2018) that the porous structure of the PEEK implant served as a mechanical interlock with the bone tissue when they evaluated the osteogenic differentiation abilities of smooth PEEK, plasma-sprayed titanium coating, and porous PEEK *in vivo* and *in vitro*. As a result, the three-dimensional customized porous structure promotes cell adhesion to the PEEK composites and thus facilitates cell growth. However, it should be noted that the use of 3D printing technology does not result in any significant changes to the bioactivity of PEEK materials. Therefore, additional modifications are necessary to enhance the bioactive properties of PEEK materials.

Nano-hydroxyapatite (nHA) is a calcium-phosphorus bone repair material with an identical chemical composition and crystal structure to human bone tissue. Its particle size usually ranges from 1 to 100 nm (Mo et al., 2023; Hoveidaei et al., 2024). nHA has strong bioactivity and biocompatibility. In the medical field, nHA has a wide range of applications, such as bone repair filler materials and implant surface coatings. It is recognized as a highly bioactive osteoinductive substance that promotes and accelerates bone regeneration (Qian et al., 2022). Zhang et al. demonstrated that nHA could successfully restrict tumor growth and encourage the regeneration of new bone by inserting porous titanium scaffolds containing nHA into bone defects in a rabbit tumor model (Zhang et al., 2019). However, adding too much nHA might weaken the mechanical characteristics of PEEK. Oladapo et al. discovered that when 3D printing PEEK materials with the addition of more than 30% calcium hydroxyapatite, the composite material showed that the aggregation in HA leads to brittle fracture and decreased mechanical properties (Oladapo et al., 2021). To address the inherent limitations in the mechanical properties of nHA, it is common practice within the medical domain to employ composite materials by incorporating nHA with other substances, such as carbon fibers (CF). Carbon fiber is a material composed of fibrous carbon crystals. It has great qualities, including lightness, outstanding strength, high modulus, and resistance to corrosion (Li Y et al., 2022). CF have been utilized for bone, bronchial, and heart valve tissue scaffolds in recent years (Tseng et al., 2021). Some researchers used sulfonation treatment of CF-reinforced PEEK to introduce hydrophilic groups to increase the material’s bioactivity. *In vitro* cellular experiments revealed that all samples had good biocompatibility and bone-enhancing activity, and *in vivo*, biosafety in rats was consistent with the results of *in vitro* experiments (Su et al., 2023). Greater surface area and improved interaction with biological tissues are provided by nanostructures and particular chemical compositions on the surface of carbon fibers. This encourages cell adhesion, proliferation, and the exchange of biological components, which increase the composite material’s bioactivity and biocompatibility (Du et al., 2022). Yang et al. successfully manufactured 10% CF/PEEK composites by using FDM. They found that the samples’ tensile strengths ranged from 50.8–135.0 MPa, and their elastic moduli were 3.5–9.2 GPa, which is close to the elastic modulus of bone tissue (Yang et al., 2021). It is possible to create PEEK composites with excellent bioactive and mechanical properties by combining CF with nHA and PEEK (Wang P et al., 2020). The main issue explored in this study is how to balance the composition of PEEK/nHA/CF composites to obtain ideal properties.

In this study, we prepared PEEK, nHA, and CF composite porous scaffolds by FDM technology and material modification to evaluate the surface morphology, mechanical properties, biocompatibility, and bone-enhancing abilities of the materials. The results demonstrated that the PEEK composites exhibited good mechanical properties and bone-enhancing effects due to the addition of nHA and CF in suitable proportions, which has a great potential for clinical translational applications.

## 2 Materials and methods

### 2.1 Preparation of PEEK composite porous scaffolds

**FIGURE 1:**
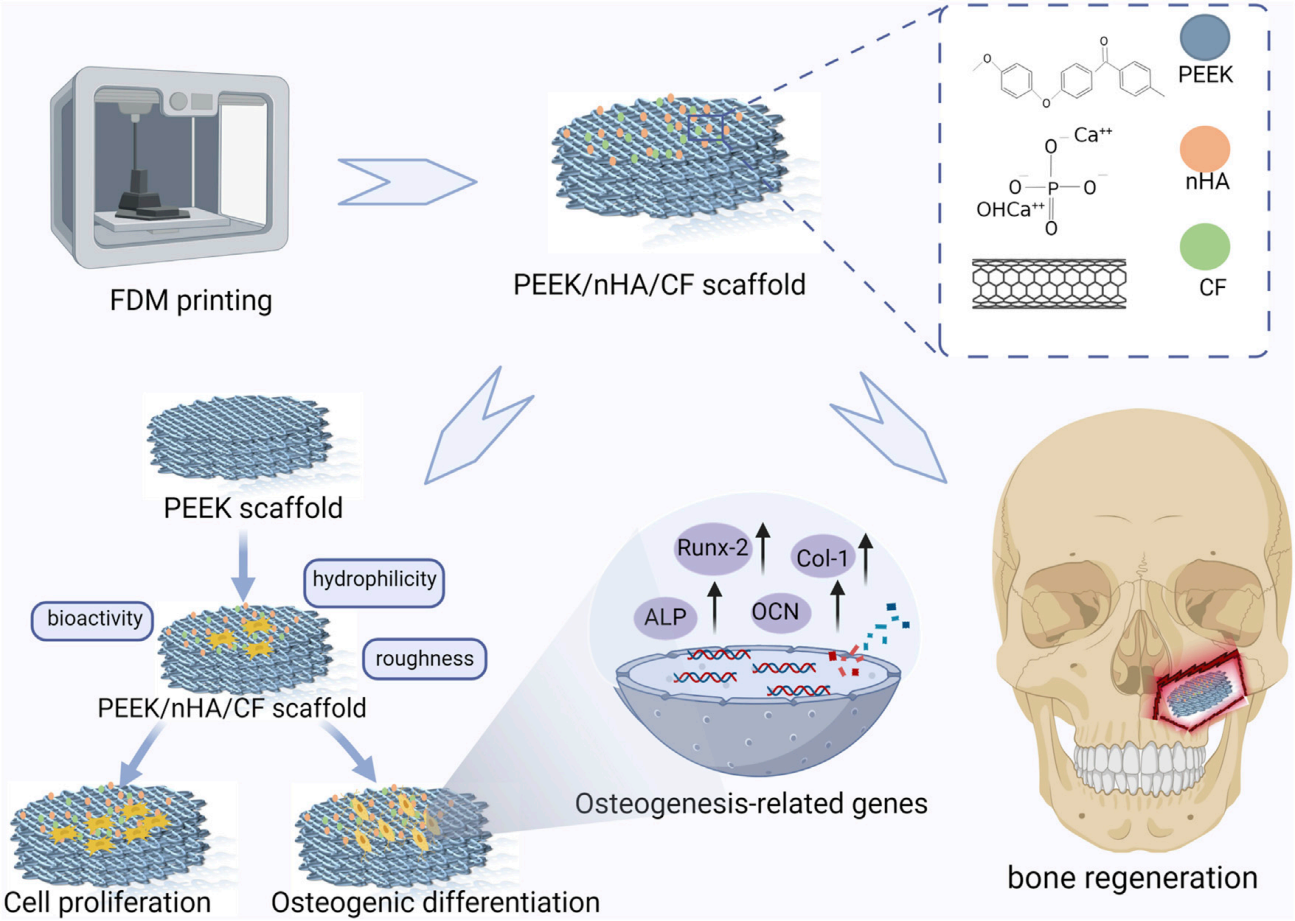
Schematic diagram of the experiment.

**FIGURE 2:**
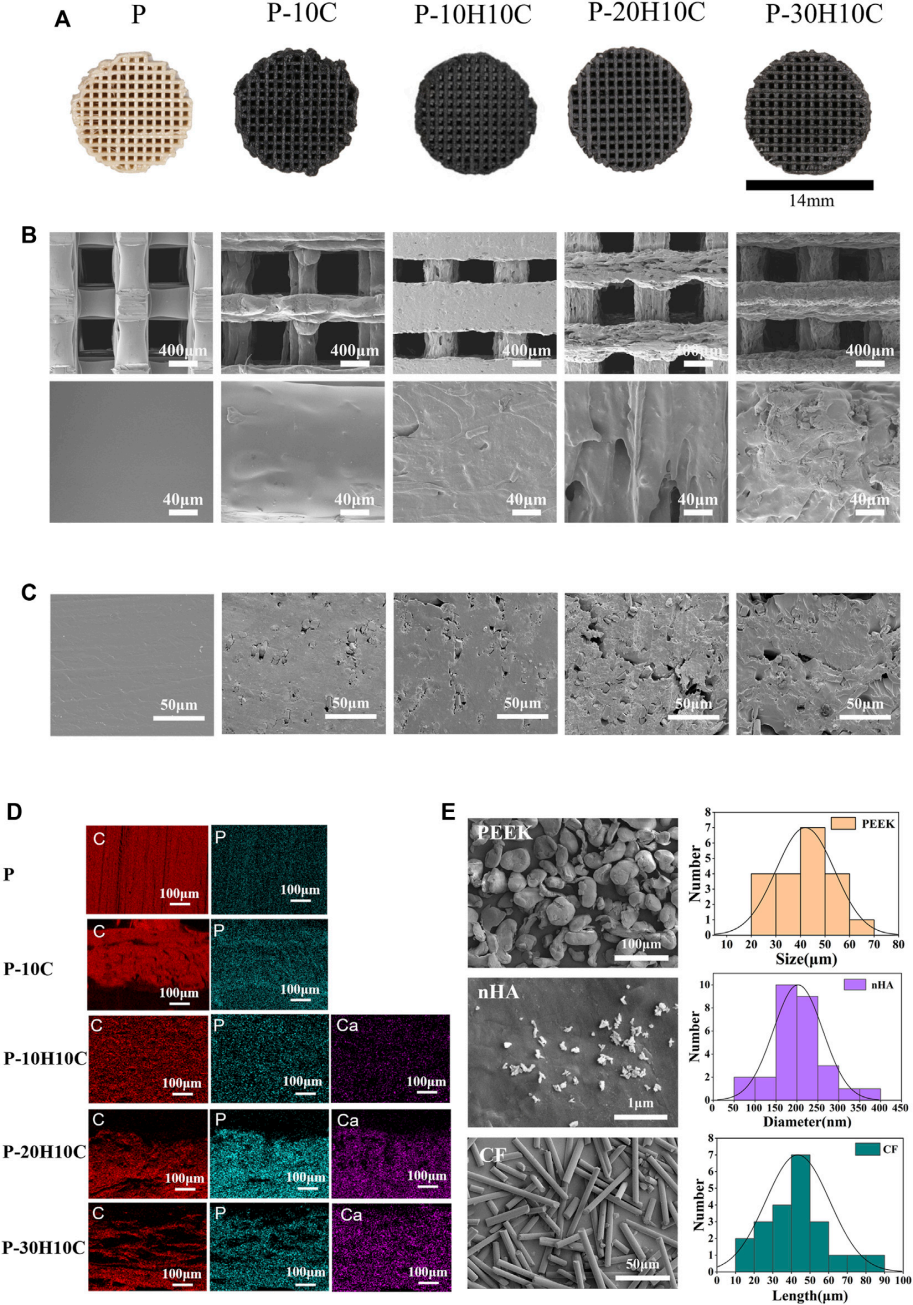
Characterization of PEEK composite scaffolds. **(A)** Visual diagram of P, P-10C, P-10H10C, P-20H10C, and P-30H10C scaffolds. **(B)** SEM surface morphology of P, P-10C, P-10H10C, P-20H10C, and P-30H10C scaffolds. **(C)** SEM of the cross-section of P, P-10C, P-10H10C, P-20H10C, and P-30H10C scaffolds. **(D)** Elemental analysis of P, P-10C, P-10H10C, P-20H10C, and P-30H10C scaffolds by EDS. **(E)** SEM and particle size distribution of PEEK, nHA, and CF powder.

As shown in Figure 1, All the specimen powders and samples were made from PEEK material and its composites from Jugao-AM Tech. (Xi’an, China). After mixing the PEEK (550 PF), nHA and CF powders with the blender in the R&D lab, all the samples were prepared into 1.75 ± 0.5 mm filaments by using a twin-screw extruder. The porous PEEK scaffold modeling was designed with 3D Max software, and the composite scaffolds samples were manufactured using the FDM. The printing parameters were 420°C print temperature, 30 mm/s print speed, 0.4 mm nozzle diameter, and 0.2 mm layer thickness for filaments that were dried at 60°C for 8 h (Table 1). The scaffolds used in each group for the *in vitro* experiments had a diameter of 1.4 mm and a height of 2 mm, as illustrated in Figure 2A. The diameter of the porous scaffolds for compression of the sample are 1.4 mm and the height is 5 mm. The tensile experiment samples were 150 mm in length and 4 mm in height (Figure 4A), following ISO 527-2: 2012 requirements (Wang P et al., 2020). As shown in Table 2, different groups of porous scaffolds were named P, P-10C, P-10H10C, P-20H10C, and P-30H10C based on the variation of PEEK, nHA, and CF content.

**TABLE 1:** Printing parameters for 3D printed PEEK composites.

Printing parameters	Value
Drying temperature for filaments (°C)	60
Printing temperature (°C)	420
Printing speed (mm/s)	30
Nozzle diameter (mm)	0.4
Layer thickness (mm)	0.2
Infill pattern	Lines

**TABLE 2:** Composition of 3D printed PEEK composites.

Samples	PEEK (wt%)	nHA (wt%)	CF (wt%)
P	100	0	0
P-10C	90	0	10
P-10H10C	80	10	10
P-20H10C	70	20	10
P-30H10C	60	30	10

### 2.2 Characterization of PEEK composite porous scaffolds

Scanning electron microscopy (SEM, TESCAN MIRA LMS, Czech) and energy dispersive spectroscopy (EDS) were used to analyze the surface micromorphology and elements of PEEK, nHA, and CF powders and the different scaffolds. The scaffold groups were then sprayed with Pt before the samples were photographed using an SEM at a 3 kV accelerating voltage for the morphology images and a 15 kV accelerating voltage for the EDS shots. PEEK, nHA, and CF powders were dispersed in ethanol, ultrasonically dispersed for 20 min, dropped on silicon wafers, dried naturally, and the surface morphology was observed by SEM. The PEEK composite scaffold’s distinctive peaks were observed using X-ray diffraction (XRD, Rigaku MiniFlex600). We explored the special functional groups of the PEEK composite scaffolds using Fourier infrared spectroscopy (FTIR; Thermo Scientific Nicolet iS20) in the spectral scan range of 500–4,000 cm^−1^. The contact angles of the porous scaffolds were photographed and recorded using an optical contact angle meter (Biolin Scientific Co., Ltd., Sweden). The surface roughness of the porous scaffolds was measured using atomic force microscopy (AFM, Dimension Icon, America). Thermogravimetric analysis (TGA, TGA55, America) was performed on a Netzsch thermal analyzer TG, and the samples were examined under an air atmosphere at a constant rate of 20°C min^−1^ in the scanning range of room temperature to 900°C with an empty aluminum pan as a reference.

### 2.3 Mechanical properties testing of PEEK composite samples

Tensile samples and 3D printed porous scaffolds were tested and analyzed in a universal testing machine (MTS Systems Ltd., China) at room temperature. The tensile speed of the samples is at a speed of 5 mm/s. Tensile strength was computed from the final data, and a stress-strain curve was shown. PEEK porous scaffolds of the compression speed are set at 0.5 mm/min. The load-displacement curve is recorded automatically, and the stress-strain curve is plotted based on the data obtained.

### 2.4 Cytocompatibility assays

Essentially, bioactivity refers to the creation of a layer like that of HA on the surface of a substance, which predicts its adherence to bone. For the prepared 3D printed porous scaffolds, biological activity was evaluated after immersion for 7 days in Simulated Body Fluid (SBF, Solarbio). The scaffolds were gently rinsed with distilled water and dried overnight at 60°C. The precipitation of apatite particles formed on the surface of the scaffold was observed by SEM. In addition, MC3T3-E1 cells were evaluated for their biocompatibility and capacity for osteogenic differentiation with different scaffolds. In a cell incubator at a culturing temperature of 37°C in an atmosphere of 5% CO_2_ and 95% humidity, cells were incubated in a complete medium (Dulbecco’s modified Eagle medium containing 10% fetal bovine serum and 1% penicillin and streptomycin). The osteogenic medium was prepared for the osteogenic differentiation assessments by adding 10 mM sodium glycerol-phosphate, 50 μg/mL L-ascorbic acid, and 10 nM dexamethasone medium to the above mediums. In addition, the scaffolds in each group were washed with acetone, ethanol, and sterile deionized water, sterilized in an autoclave, and dried before cell experiments (He et al., 2019).

#### 2.4.1 Cell biocompatibility on the porous scaffolds

MC3T3-E1 cells were seeded at a density of 1×10^5^ in 24-well plates, then added to different groups of PEEK porous scaffolds for indirect incubation, and cultivated for 72 h. Cells were washed twice with PBS, shielded from light, and added with Live/Dead Cell Double Staining Kit (Meilunbio), then incubated at 37°C for 30 min before being photographed with an inverted fluorescent microscope.

In the cell adhesion experiments, MC3T3-E1 cells were directly cultured on five groups of PEEK porous scaffolds for 72 h. Following this, the scaffolds were rinsed twice with PBS, fixed for 3 h with 2.5% glutaraldehyde, dehydrated by gradient with different concentrations of ethanol, and dried in a vacuum drying oven. SEM photographs of cells adhering to the surface of the scaffolds were captured after the groups of scaffolds were sprayed with Pt.

The cell proliferation assay was assessed by CCK-8 assay. First, 0.1 g/mL (ISO 10993- part 12) of the scaffold solution was extracted after 48 h in the medium. After that, 96-well plates containing MC3T3-E1 (5×10^3^ cells/well) were incubated with the cells for 24 h. Different groups of extracts were added to the MC3T3-E1 cells in the plates after 24h, 48h, and 72 h. 10μL of the CCK-8 solution was added to each well, which was then incubated for 1 h at 37°C in a cell culture incubator. At last, the optical density (OD) at 450 nm was obtained using a spectrophotometer (HBS-1069A).

#### 2.4.2 Osteogenic differentiation of cells on the porous scaffolds

By evaluating ALP concentration, the early osteogenic activity of various groups of porous scaffolds indirectly grown with MC3T3-E1 cells for 7 and 14 days was evaluated. Cell suspensions were seeded onto 24 culture plates at a density of 1×10^5^ cells/well and put on different scaffolds for indirect culture, with new fluids every 2 days. After 7 and 14 days of culture in osteogenic medium, the scaffolds were removed, washed twice with PBS, fixed with 4% paraformaldehyde for 30 min, and stained with ALP using BCIP/NBT staining kit, and the cell staining was observed through a microscope and photographed for recording. After then, a quantitative ALP analysis was carried out.

To assess the level of mineralization of the scaffolds, the scaffolds were incubated indirectly with the cells for 14 and 21 days, and after fixation with 4% paraformaldehyde, the calcified nodules were stained with alizarin red staining solution to assess the ability to mineralize at a later stage. An inverted microscope was used to take pictures and record the staining pattern of the cells. The calcium nodules in each well were dissolved with 1 mL of cetylpyridinium chloride, and stayed for 30 min at room temperature. The OD value was measured at 562 nm using an enzyme marker.

To analyze the expression of osteogenesis-related genes, RNA was extracted from different groups of MC3T3-E1 cells after 7, 14, and 21 days of osteogenic induction using an RNA extraction kit, and then reverse-transcribed into cDNA using the Prime ScriptTM RT Master Mix Kit (Takara). TB Green^®^ Premix Ex TaqTM II (Takara) and CFX 96 real-time quantitative PCR were used for evaluating the mRNA expression levels of Runt-related transcription factor 2 (Runx2), osteocalcin (OCN), alkaline phosphatase (ALP), collagen-1 (Col-1) and GAPDH in real-time. The sequences of primers are shown in Table 3.

**TABLE 3:** Primer sequences for qRT-PCR.

Rat gene	Forward primer (5′–3′)	Reverse primer (5′–3′)
Runx-2	CCT​CCA​GCA​TCC​CTT​TCT​T	CCTTTTCCCTCCTTGCCT
OCN	ACC​ATC​TTT​CTG​CTC​ACT​CTG​CT	CCT​TAT​TGC​CCT​CCT​GCT​TG
ALP	GGC​AAA​GAG​GGA​GCT​AGA​A	ATGGCCGTGCAGATGTA
Col-1	GAC​ATG​TTC​AGC​TTT​GTG​GAC​CTC	GGG​ACC​CTT​AGG​CCA​TTG​TGT​A
GAPDH	TCA​CCA​TCT​TCC​AGG​AGC​GAG​AC	TGA​GCC​CTT​CCA​CAA​TGC​CAA​AG

### 2.5 Statistical analysis

All experimental data were statistically analyzed using GraphPad Prism (version 9.0) software. One-way analysis of variance (ANOVA) was used for multiple group comparisons and Tukey’s multiple comparison test was used between group comparisons. *P* < 0.05, differences were considered statistically significant.

## 3 Results and discussion

### 3.1 Characterization of PEEK composite scaffolds

In the conducted experiment, a variety of composite PEEK scaffolds were fabricated through the utilization of FDM technology. These scaffolds consisted of diverse combinations of nHA, CF, and PEEK, denoted as P, P-10C, P-10H10C, P-20H10C, and P-30H10C, as outlined in Table 2. As depicted in Figure 2A, the utilization of FDM technology in the fabrication of the hybrid stent structure results in the formation of an intricate three-dimensional configuration characterized by a grid-like structure composed of minute pores and channels. Figure 2E shows the surface morphology of PEEK, HA, and CF powders. PEEK powder has an irregular shape with a particle size of 20–70 μm. nHA has a needle-like morphology with a particle size of 50–400 nm, and agglomerates appear on the surface of the material. CF has a rod-like distribution with a length of 10–90 μm. The SEM pictures, when magnified by factors of 100, revealed the presence of a highly porous and linked network structure in five distinct sample groups (Figure 2B). Further, these photos exhibited intricate surface textures and intricate features. The results of the SEM analysis indicated that the surface of sample P exhibits a smooth texture. Nevertheless, it was observed that sample P-10C exhibit the existence of CF protrusions. The other three scaffold groups, on the other hand, demonstrated that with the addition of nHA, particles or protrusions developed on the scaffold surface, affecting the surface roughness. The average pore size of the hybrid stent was determined using ImageJ software, as depicted in Figure 2B. The P, P-10C, P-10H10C, P-20H10C, and P-30H10C obtained measurements indicated that the pore size ranges from 632.97 ± 21.85 μm, 637.25 ± 44.51 μm, 471.63 ± 99.66 μm, 560.23 ± 70.07 μm, and 554.43 ± 44.91 μm. The pore size data that has been acquired is of utmost importance in understanding the characteristics of the scaffold. This is because the pore size plays a critical role in facilitating vascularization throughout the process of tissue regeneration, as well as influencing cell proliferation and migration (Qian et al., 2023a). According to initial research findings, it has been shown that a hole size of approximately 400–600 μm is deemed optimal for facilitating complete mineralized bone development and vascularization, hence promoting the process of osteogenesis (Qin et al., 2022). Figure 2C shows the cross section of the porous scaffolds. The surface of the scaffolds is rougher except for group P, where the surface is dense and smoother and a cross-section of rod-like carbon fibers can be seen embedded inside the scaffolds. The distribution of white nHA particles can be seen for P-10H10C, P-20H10C, and P-30H10C.

The element distribution of five groups of PEEK scaffolds can be depicted by the process of element mapping in an EDS diagram (Figure 2D). Both PEEK and CF contain a significant amount of carbon (C) and small amounts of phosphorus(P). As a result, the materials P and P-10C exhibit regions with high carbon content. In the case of mixed PEEK incorporating nHA, the EDS analysis may reveal that calcium (Ca) and phosphorus (P) are the predominant element, alongside carbon. It is worth noting that Ca and P serves as the primary constituent of calcium phosphate (CaPO_4_) inside the nHA composition.

**FIGURE 3:**
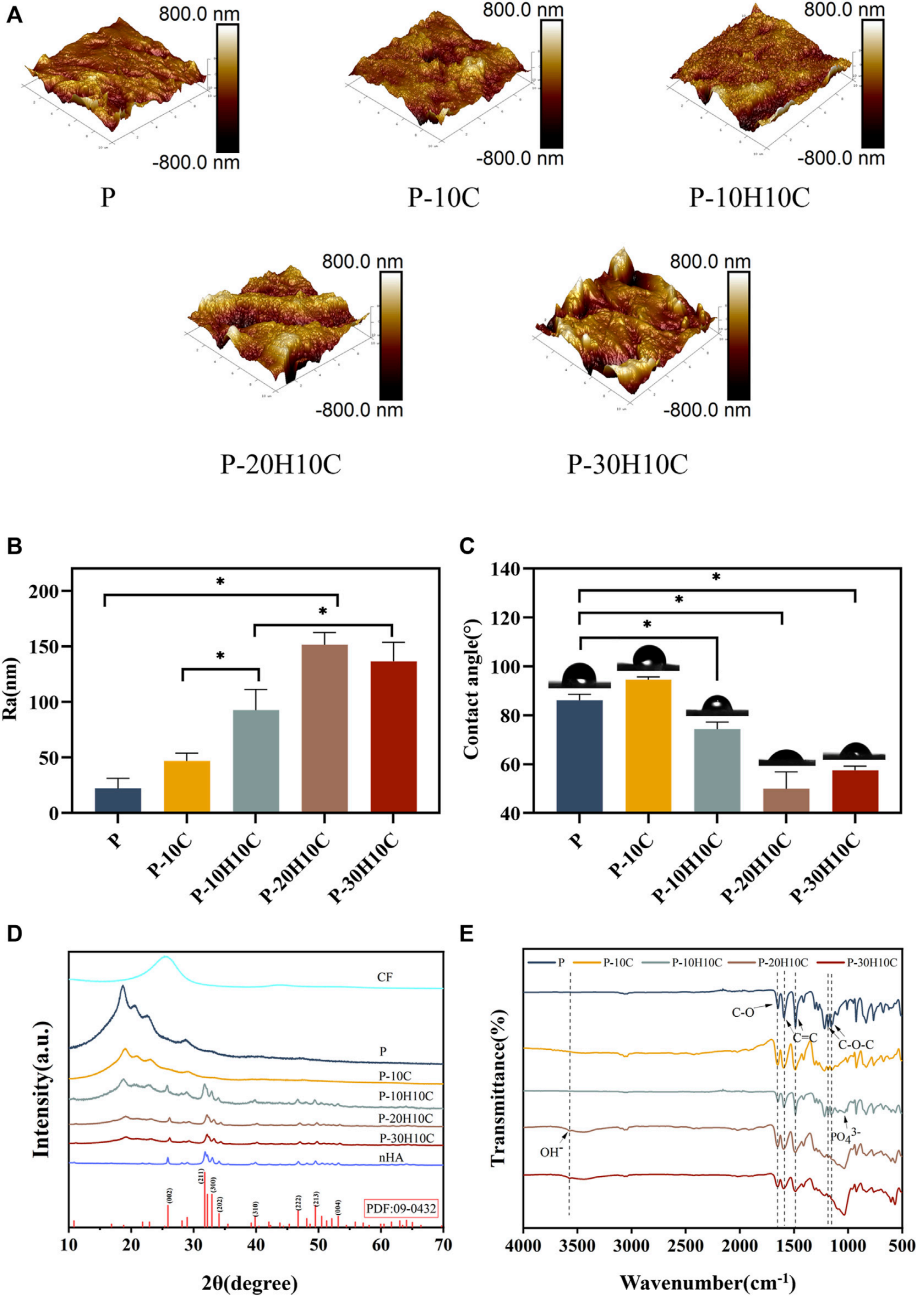
Characterization of PEEK composite scaffolds. **(A)** AFM morphology of P, P-10C, P-10H10C, P-20H10C, and P-30H10C scaffolds. **(B)** Roughness analysis of different PEEK porous scaffolds (*p < 0.05). **(C)** Analysis of water contact angles for different PEEK porous scaffolds (*p < 0.05). **(D)** XRD analysis of P, P-10C, P-10H10C, P-20H10C, and P-30H10C scaffolds and nHA and CF powder. **(E)** FTIR analysis of P, P-10C, P-10H10C, P-20H10C, and P-30H10C scaffolds.

The XRD patterns of CF powder, HA powder, and five groups of scaffolds are shown in Figure 3D. There were about four reflection peaks corresponding to the PEEK crystal structure, located at 18.8°, 20.7°, 22.7°, and 28.8°, respectively. The incorporation of CF did not influence the crystal structure of PEEK. In P-10H10C, P-20H10C, and P-30H10C, nHA diffraction peaks were seen. The typical modes of the nHA characteristic peaks include (002), (211), (300), (222), and (213) (Liu et al., 2019; Cui et al., 2021). The positions of these peaks coincide with the data of TCPDS Card No. 09-0432 on the standard peaks of HA. It was demonstrated that pure HA was present in P-10H10C, P-20H10C, and P-30H10C, indicating that nHA was successfully incorporated into the PEEK composite porous scaffolds (Zheng et al., 2022).

**FIGURE 4:**
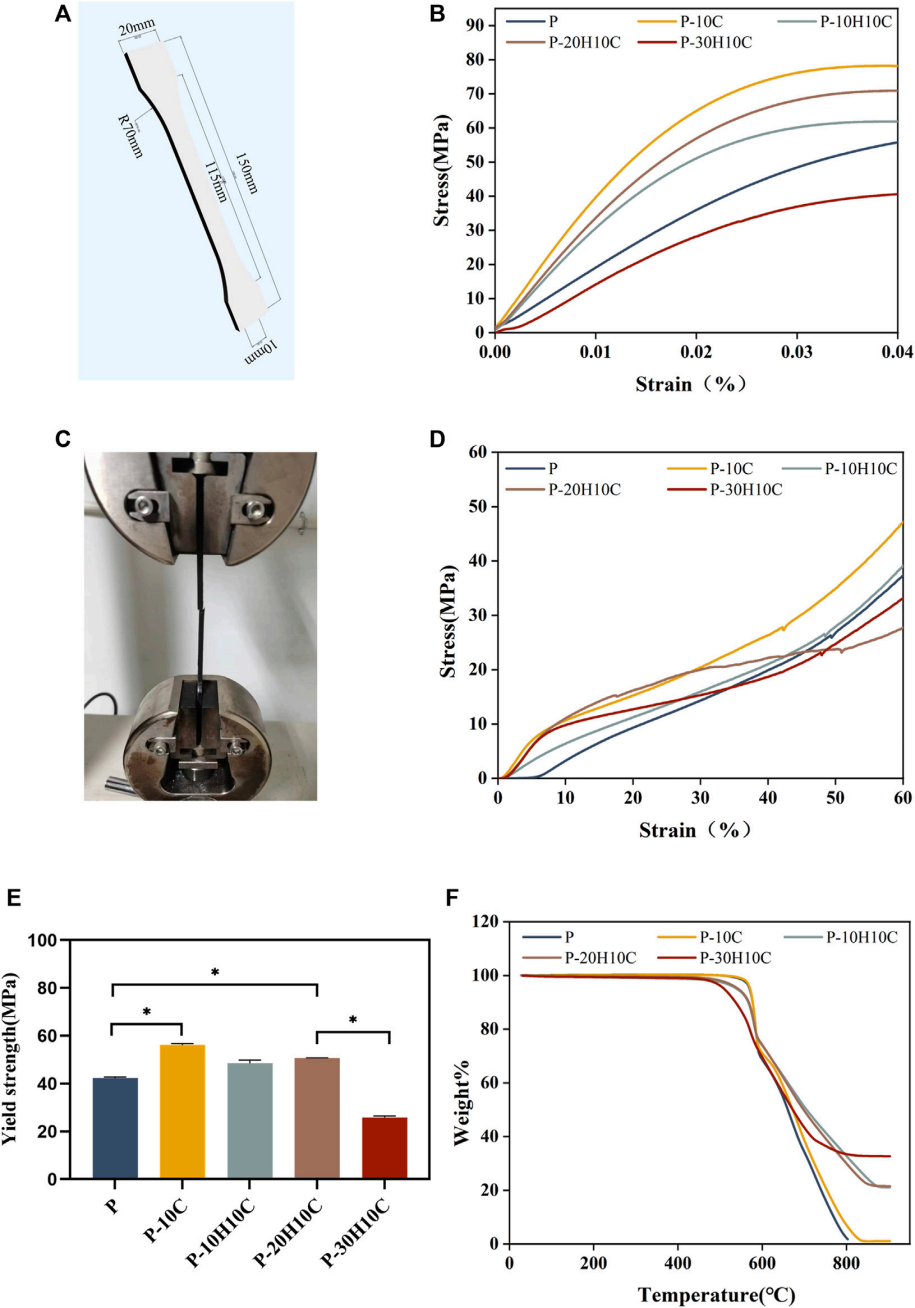
Analysis of the mechanical properties of PEEK composites and thermal properties. **(A)** 3D modeling of PEEK tensile samples. **(B)** Stress-strain curves for different groups of tensile samples. **(C)** Sample stretching process. **(D)** Stress-strain curves for different groups of compression samples. **(E)** Yield strength of different groups (*p < 0.05). **(F)** TGA curves of P, P-10C, P-10H10C, P-20H10C, and P-30H10C scaffolds.

The FTIR spectra labeled in Figure 3E demonstrate the characteristic absorption peak of PEEK in all five composite scaffolds. It showed the carbonyl stretching vibration of C=O at a peak at 1,646 cm^−1^. The peaks at 1,598 cm^−1^ and 1,501 cm^−1^ are caused by vibrations in the C=C benzene plane. As the nHA content increased, the intensity of the PO_4_^3-^ and OH^−^ peaks increased (Zheng et al., 2022; Zheng et al., 2021). The peak of the P-10H10C, P-20H10C, and P-30H10C composite scaffolds was caused by vibrations at 1,047 cm^−1^(PO_4_^3-^) and 3,575 cm^−1^ (OH^−^). The thermal stability of the 3D printed porous scaffolds was analyzed by TGA. The TGA thermal spectra of each group of scaffolds are given in Figure 4F. When the temperature exceeded 470°C, the materials of each group began to decompose. The initial decomposition temperature of P and P-10C was 562°C. The initial decomposition temperature of P-10H10C, P-20H10C, and P-30H10C was 470°C. The initial decomposition temperature of P-10H10C, P-20H10C, and P-30H10C was 470°C. At 800°C, the residual weight percentage of P group scaffolds was 1.73%; at 900°C, the residual weight percentage of P-10C was 7.34%; and the residual weight percentages of scaffolds after addition of nHA were 21.05% for P-10H10C, 21.86% for P-20H10C, and 32.94% for P-30H10C, respectively. The decomposition temperature of the porous scaffolds was greater than the set temperature of 420°C for 3D printing, indicating that the prepared scaffolds did not decompose during the printing process and had good thermal stability.

As shown in Figures 3A, B, we analyzed the roughness of the material surface by AFM. The surface of the P scaffold exhibited a smooth texture. However, upon the addition of 10% CF powder, an internal network structure was generated within the scaffold. This is attributed to the interaction between PEEK and CF during the mixing process. Consequently, the surface micro texture of the P-10C scaffold was enhanced, accompanied by a little increase in roughness. Nevertheless, the incorporation of nHA particles could lead to the formation of particle distribution inside scaffold materials, resulting in various interactions between nHA particles and PEEK. The incorporation of nHA into a PEEK scaffold resulted in alterations in surface roughness, the impact might vary depending on the quantity of nHA added. The addition of nHA in P-10H10C, P-20H10C, and P-30H10C composites led to a moderation of the interfacial tension and an enhancement in material fluidity, as compared to P-10C alone. The increased roughness of P-20H10C might be attributed to the elevated concentration of nHA particles, which have the tendency to deposit or aggregate on the surface of the scaffold. This deposition and aggregation process leads to the formation of irregular protrusions, thus resulting in an augmented roughness of the scaffold surface. Nevertheless, the potential explanation for the decreased roughness seen in P-30H10C could be attributed to the presence of nHA particles, which have an impact on the unrestricted motion of fluid molecules and thus lead to a reduction in surface roughness (Wang et al., 2022).

The hydrophilicity analysis of the material is shown in Figure 3C. As the amount of nHA in the material increases, the hydrophilic angle of the surface decreases. P-20H10C exhibits the highest hydrophilicity in comparison to PEEK. It is possible to determine the reason for this phenomenon because, when exposed to water, nHA may form a hydrophilic coating on the material’s surface (Jang et al., 2020). This hydrophilic layer can lower the material’s surface tension, facilitating water diffusion on the material’s surface and enhancing the material’s hydrophilicity (Durham et al., 2016). At the same time, the hydrophilic layer can absorb the nearby water molecules, resulting in surface wetting and enhancing the material’s hydrophilicity even more. According to earlier research, the calcium and phosphate ions on the surface of nHA may induce biological reactions and cell signaling by attaching to receptors on cell membranes. These signaling systems can increase cell differentiation, proliferation, and secretory activity, which promotes cell adhesion and proliferation (Zhang et al., 2022; Zhong et al., 2022).

### 3.2 Mechanical properties of different PEEK composites

Good mechanical properties are essential for human implant materials. The analysis of the mechanical properties of each group of tensile samples was shown in Figure 4. The porous structure of the 3D printing reduced the elastic modulus of the PEEK material compared to prior research (Wang P et al., 2020). However, Figure 4B demonstrated that P-10C has the best mechanical qualities and the greatest tensile strength. In comparison to P, the yield strength of P-10C increased from 42.35 ± 0.42 MPa to 56.15 ± 0.54 MPa (Figure 4E). It is important to note that the mechanical properties of PEEK/CF were less affected by the addition of just a small portion of nHA. The results of sample compression are consistent with the results of tensile experiments (Figure 4D). However, as the nHA content increased to 30%, the tensile and yield strengths of the composites declined. This might be related to the binding mechanisms of PEEK and nHA. It has been demonstrated that adding too much nHA can cause stress concentration and brittle fracture at the interface between both materials (Kang et al., 2022). When the nHA particle content was excessively high, the interaction force between particles increased, which led to particle aggregation and accumulation (Kim et al., 2020; Hwangbo et al., 2022). This phenomenon results in a concentration of interface tensions between the nHA particles and the PEEK matrix, weakening the material’s strength and toughness.

### 3.3 Cell biocompatibility experiments with different porous scaffolds

**FIGURE 5:**
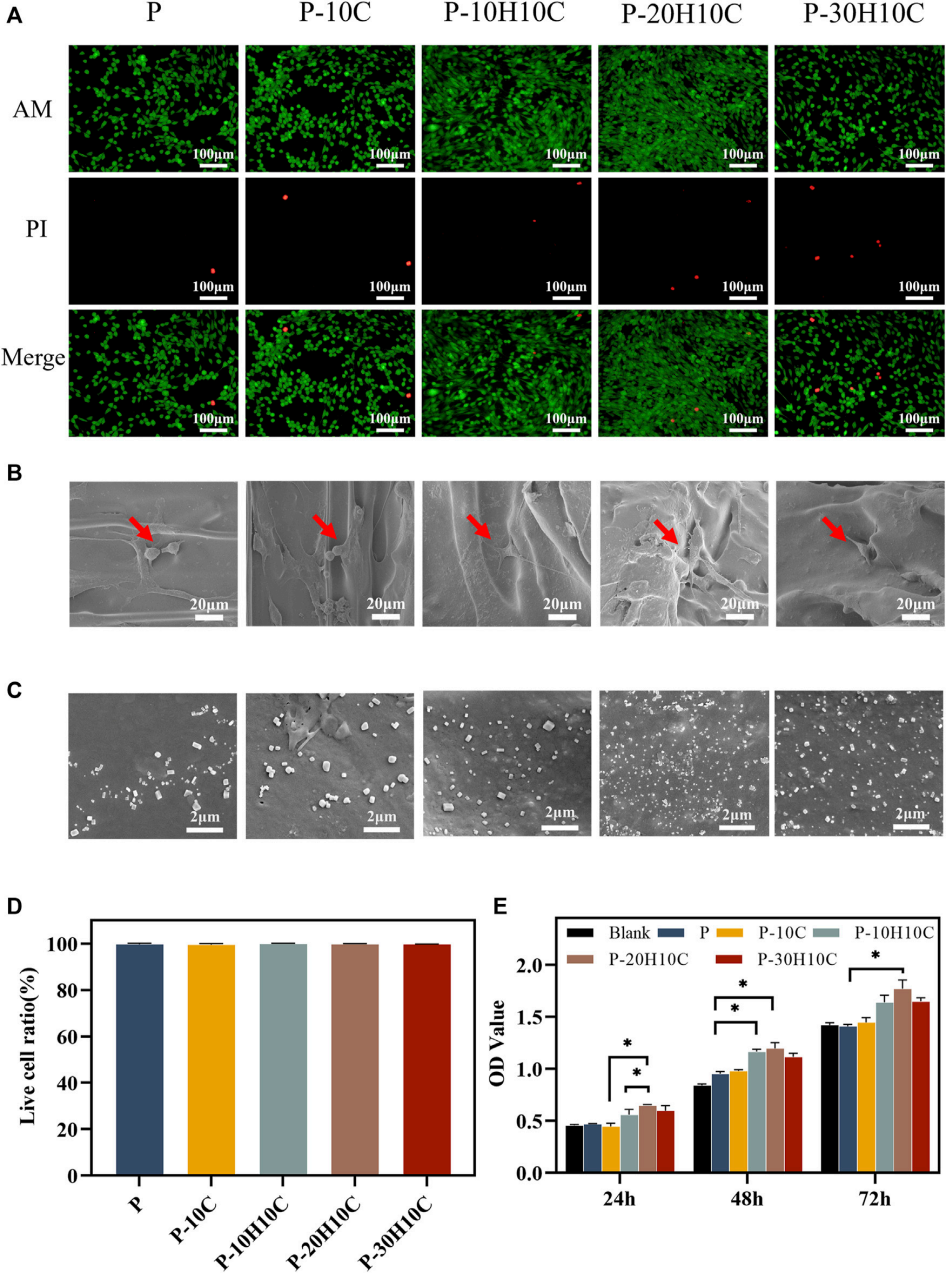
Cell biocompatibility experiments with different PEEK porous scaffolds. **(A)** Live dead cell staining of P, P-10C, P-10H10C, P-20H10C, and P-30H10C. **(B)**The SEM image of cells adhering to different porous scaffolds. **(C)** SEM micrographs of samples’ surface after immersion in SBF for 7 days: P, P-10C, P-10H10C, P-20H10C, and P-30H10C scaffolds. **(D)** Live cell ratio analysis. **(E)** Cell proliferation capacity in different scaffolds by CCK8 assay (*p < 0.05).

In the cell live-dead staining experiment, after incubating the cells indirectly with the scaffolds for 3 days, a large number of live cells with green fluorescence and a small number of dead cells with red fluorescence could be seen in all groups of materials (Figure 5A). The ratio of living to dead cells showed no statistically significant variation (Figure 5D), demonstrating the PEEK composite scaffolds’ high biocompatibility, and the cells exhibited activity on the surface of the scaffold and demonstrated a high rate of proliferation.

The SEM images of cell adhesion experiments revealed that MC3T3-E1 cells adhered to the scaffold’s surface in all groups (Figure 5B). Different scaffolds displayed various patterns of cell adhesion. The majority of the cells in P and P-10C were connected in a spherical pattern. The cells in P-10H10C and P-20H10C showed filamentous pseudopods, which expanded in quantity and spread across the surface of the materials. The main reason was that adding nHA and CF to PEEK composites could improve their surface features, making them more biocompatible and bioactive. nHA could interact with cells to foster cell adhesion because it has a microstructure and chemical composition that is close to bone tissue. Because of its high specific surface area and superior mechanical qualities, CF could offer more areas of adhesion and support, which may be helpful in cell growth and adhesion (Rodzeń et al., 2021; Zhong et al., 2021). Figure 5C shows the precipitated particles formed by each group of scaffolds after 7 days of immersion in SBF. A large distribution of precipitated material was visible on the scaffold surface after nHA was added. By incorporating HA nanoparticles into the composite scaffolds, bone regeneration was promoted, which led to greater biomineralization capacity.

The CCK-8 assay was used to measure the proliferation ability of cells after 24h, 48h, and 72 h of culture, respectively (Figure 5E). The results indicated that P and P-10C increased less rapidly than the blank group. After 72 h of culture, cells with the nHA groups exhibited higher OD values, indicating the cells’ ability to proliferate most effectively. This confirmed that the addition of nHA could improve PEEK’s biological activity. Researchers have demonstrated that nHA could significantly roughen up the surface of the material, promoting cell proliferation and osteogenic differentiation (Chen et al., 2020). It was noted in the literature that Sari constructed porous HA scaffolds by precipitating hydroxyapatite and honeycomb (HCB) together, proving the material’s excellent capacity for cell proliferation and metabolic activity (Sari et al., 2021). Furthermore, nHA released calcium ions that can impact cell signaling, the production and breakdown of extracellular matrix, and the process of cell proliferation and differentiation.

### 3.4 Osteogenic differentiation of MC3T3-E1 cells in porous scaffolds

The osteogenic differentiation of MC3T3-E1 cells on the material’s surface could demonstrate the material’s bone-enhancing capabilities. Previous research has focused on the osteogenic activity of small amounts of nHA (Chen et al., 2019). Using ALP qualitative and quantitative assays, ARS staining and quantitative analysis, and PCR experiments, we confirmed that PEEK composite porous scaffolds have great osteogenic differentiation ability. Through the utilization of ALP qualitative and quantitative assays, ARS staining and quantitative analysis, as well as PCR investigations, we have successfully validated that PEEK composite porous scaffolds exhibit a significant enhancement in the capacity to promote cellular osteogenic differentiation.

**FIGURE 6:**
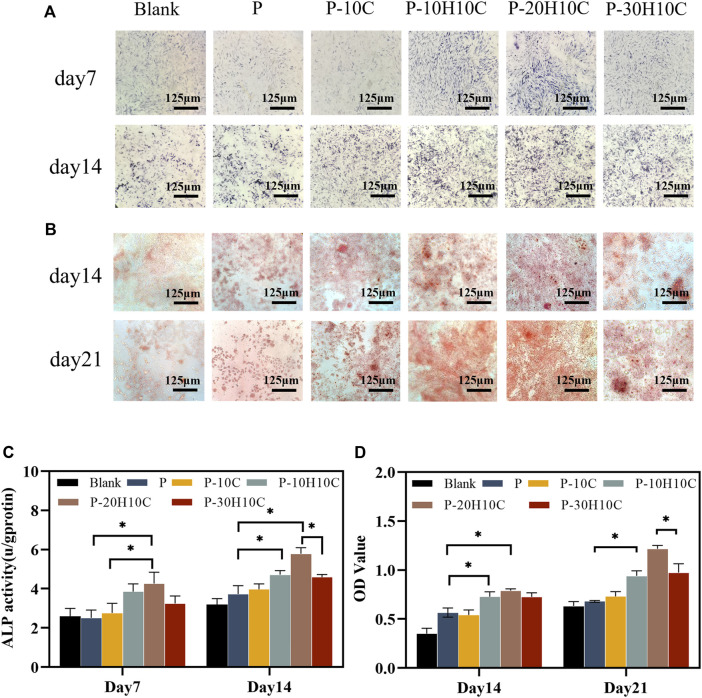
Osteogenic differentiation of MC3T3-E1 cells in porous scaffolds. **(A)** ALP staining after 7 and 14 days of cell culture. **(B)** ARS staining after 14 and 21 days of cell culture. **(C)** Statistical analysis of ALP staining at 7 and 14 days (*p < 0.05). **(D)** Statistical analysis of ARS staining at 14 and 21 days (*p < 0.05).

We cultivated MC3T3-E1 cells for 7 and 14 days to evaluate their ability to differentiate into osteoblasts. The MC3T3-E1 cells were subjected to osteoblast induction media for a duration of 7 and 14 days in order to assess their early-stage osteoblast differentiation potential. At day 7, the difference in ALP activity between P-10H10C and P-20H10C was not statistically significant, but they were both higher than that of the blank group and the P group (Figures 6A, C). After 14 days, ALP staining revealed that the surface colors of the early osteogenic differentiation of MC3T3-E1 cells in the porous scaffolds in the various groups were darker than those of the cells at day 7. P-20H10C was more apparent in cells (Figure 6A).

ARS staining revealed that MC3T3-E1 cells had late osteogenic differentiation in different porous scaffolds (Figure 6B). At 14 days, only a small amount of calcium nodule deposition was observed in the pore plates. At 21 days, a significant increase in calcium nodule deposition was observed with the increase of nHA content. ARS Quantitative analysis verified this (Figure 6D). By using the FDM technology, Babilotte combined nHA with medical-grade poly (lactic-co-glycolic) acid (PLGA) and discovered that the material with the additional nHA had a greater level of cell mineralization and a significant increase of calcium nodule deposition (Babilotte et al., 2021). This further demonstrated nHA’s capacity to promote matrix mineralization and osteoblast development.

**FIGURE 7:**
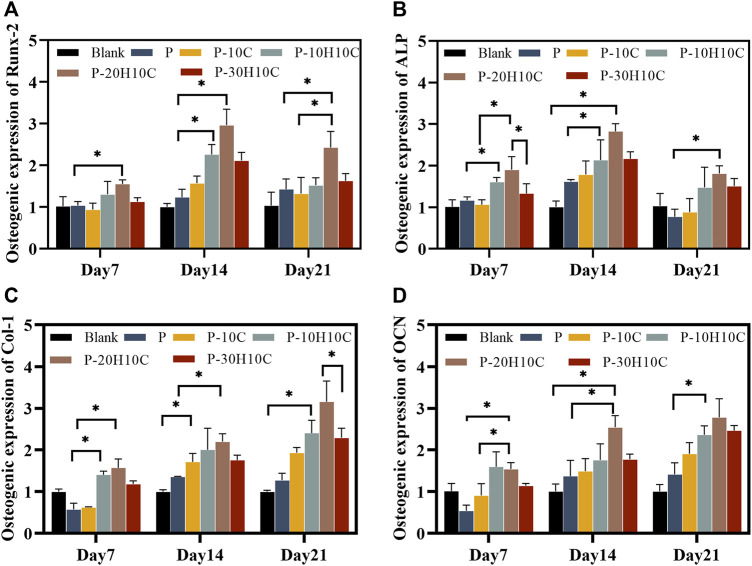
Osteogenic gene expression of MC3T3-E1 cells in porous scaffolds at 7 days, 14 days, and 21 days. **(A)** Osteogenic expression of Runx-2. **(B)** Osteogenic expression of ALP. **(C)** Osteogenic expression of Col-1. **(D)** Osteogenic expression of OCN. (*p < 0.05).

We assessed the expression of osteogenic genes in the osteogenic differentiation of MC3T3-E1 cells. Runx-2 and ALP were indicators of early osteoblast differentiation. Runx-2 was primarily involved in the transcription of osteogenic genes (Pérez-Campo et al., 2016). The expression levels of Runx-2 in each scaffold group on days 7, 14, and 21 are shown in Figure 7A. The expression levels of P-10H10C and P-20H10C were higher than P, which was statistically different. ALP primarily participates in phosphate hydrolysis processes and encourages bone matrix mineralization (Sardiwal et al., 2013). ALP activity is represented as a biomarker of bone formation; it primarily participates in phosphate hydrolysis processes and encourages bone matrix mineralization. It can also indicate the stage of osteoblast development and activity. The expression of the ALP gene reached its highest level on day 14, and the expression decreased on day 21 (Figure 7B). Col-1 is a protein that predominantly promotes osteoblast adhesion and differentiation, facilitating bone repair (Bou Assaf et al., 2019). At day 21, the cellular expression of the P-10H10C, P-20H10C, and P-30H10C scaffolds was higher than that of P, which might be attributed to the inclusion of nHA, which roughened the surface of the scaffolds (Figure 7C). This might be related to the ability of nHA to offer a surface for cell adhesion and penetration, giving MC3T3-E1 cells an environment favorable for cell adhesion, proliferation, and differentiation. In this situation, Col-1 can be generated more frequently by MC3T3-E1 cells. By increasing collagen matrix synthesis, nHA indirectly influences Col-1 expression. However, the large amount of agglomeration of 30% nHA in the composites restricts the interaction of cells with the material and thus reduces the osteogenic differentiation ability of the cells. In addition, OCN is a marker indicating the maturation stage of osteoblasts (Li F et al., 2022). It is crucial for maintaining the mechanical characteristics of bone and bone density and was primarily engaged in the mineralization process of the bone matrix. As the amount of time increased, OCN expressed itself proportionately (Figure 7D). The expression of osteogenic genes like Col-1 and OCN was indirectly impacted by nHA because it enhanced Runx-2 expression and activity, which in turn encouraged the differentiation of MC3T3-E1 cells toward osteogenesis. These findings supported the ALP and ARS results, which indicate that P-10H10C, P-20H10C, and P-30H10C all enhance osteogenic differentiation, with P-20H10C having the strongest effects. Therefore, it could be assumed that the addition of nHA particles stimulated the expression of relevant osteogenic genes and promotes osteoblast mineralization.

## 4 Conclusion

In this research, we successfully prepared PEEK/nHA/CF composite porous scaffolds by FDM technology. It was shown that the composite porous scaffolds had excellent mechanical properties, good biocompatibility, and bone differentiation ability. Compared with the unmodified porous PEEK scaffolds, P-20H10C showed higher surface roughness, obviously high hydrophilicity, and excellent performance in cell proliferation and osteogenic differentiation. Our mechanical property analysis also showed that the addition of 10% CF effectively balanced the adverse effect of nHA on the mechanical properties. In summary, the P-20H10C scaffold had excellent mechanical properties and bioactivity. As a result, the porous structure design and surface modification of PEEK materials greatly improved the osteointegration ability of PEEK materials. It provides a superior approach for biomedical materials in bone defect repair and has good potential for clinical translation.

## Data Availability

The original contributions presented in the study are included in the article/Supplementary Material, further inquiries can be directed to the corresponding authors.
